# Cognitive stimulation and occupational therapy for *delirium* prevention

**DOI:** 10.5935/0103-507X.20170034

**Published:** 2017

**Authors:** Eduardo Tobar, Evelyn Alvarez, Maricel Garrido

**Affiliations:** 1 Departamento de Clínica Médica, Campus Norte, Facultad de Medicina, Universidad de Chile - Santiago, Chile.; 2 Unidade de Terapia Intensiva, Hospital Clínico, Universidad de Chile - Santiago, Chile.; 3 Departamento de Ciências da Saúde, Escola de Terapia Ocupacional, Universidad Central de Chile - Santiago, Chile.; 4 Departamento de Medicina Física e Reabilitação, Hospital Clínico, Universidad de Chile - Santiago, Chile.

**Keywords:** Delirium/prevention & control, Occupational therapy

## Abstract

*Delirium* is a relevant condition in critically ill patients with long-term impacts on mortality, cognitive and functional status and quality of life. Despite the progress in its diagnosis, prevention and management during the last years, its impact persists being relevant, so new preventive and therapeutic strategies need to be explored. Among non-pharmacologic preventive strategies, recent reports suggest a role for occupational therapy through a series of interventions that may impact the development of *delirium*. The aim of this review is to evaluate the studies evaluating the role of occupational therapy in the prevention of *delirium* in critically ill patient populations, and suggests perspectives to future research in this area.

## Abstract (pt)

O *delirium* é uma condição importante em pacientes críticos, com impactos em longo prazo em termos de mortalidade, condição cognitiva e funcional, e qualidade de vida. Apesar do progresso ocorrido nos anos recentes em seu diagnóstico, prevenção e tratamento, seu impacto continua relevante, de forma que é necessário explorar novas estratégias de prevenção e tratamento. Dentre as estratégias preventivas não farmacológicas, relatos recentes sugerem o papel da terapia ocupacional por meio de uma série de intervenções que podem ter impacto no desenvolvimento do *delirium*. O objetivo desta revisão é avaliar os estudos que discutem o papel da terapia ocupacional na prevenção do *delirium* em populações de pacientes críticos, além de sugerir perspectivas para pesquisas nesta área.

## INTRODUCTION

Intensive care unit (ICU) *delirium* is a relevant condition for intensive care patients and professionals. This complication is relevant due to its high incidence and its potential to affect patient outcomes in the short and long term.^(1,2)^ Different pharmacological and non-pharmacological strategies have been evaluated for the prevention and treatment of ICU *delirium,* with heterogeneous results to date.^(3,4)^ New strategies to limit the impact of this condition are necessary despite advances in the field.^(5)^

Recently, some studies have explored the role of occupational therapy (OT) in the ICU alone or more frequently as part of the rehabilitation team.^(6,7)^ Some of these studies have explored *delirium* as a principal or secondary outcome. In view of these recent studies, our objective was to review the literature exploring the role of OT in the ICU, particularly in the area of *delirium* prevention.

### Key concepts in occupational therapy

According to the World Federation of Occupational Therapy (www.wfot.org), OT is the art and science of enabling engagement in everyday living through occupation. The primary goal of OT is to enable people to participate in the Activities of Daily Living (ADL). Occupational therapy interventions directly affect the person via sensorial, motor or cognitive interventions and/or the environment with physical and social interventions. These interventions targeting different components of health are intended to improve functional performance and social inclusion.^(8)^

Occupational therapy has shown physical, cognitive, and functional benefits for patients with a variety of health conditions. In adult populations, stroke rehabilitation guidelines recommend OT to improve independence with basic ADL (BADLs).^(9-11)^ In dementia, OT has been shown to improve behavioral and functional scores, slow disease progression, and decrease caregiver burdens.^(12-14)^ There is also moderate evidence that OT can improve traumatic brain injury rehabilitation and chronic pain management.^(15-17)^

Occupational therapy-based cognitive rehabilitation for these pathologies typically includes sensory stimulation, cognitive training (e.g., attention, memory, and executive functions), and caregiver/family education. Repetitive exercises and tasks specific to practice BADLs (i.e., grooming, dressing, and bathing) are used to improve physical functions, and environmental modification is applied to facilitate cognitive and functional performance.

### Occupational therapy in the intensive care unit

Therapeutic advances have increased survival for patients admitted to the ICU. However, ICU patients with severe pathologies and/or prolonged ICU stays have a higher risk for long-term neuromuscular, cognitive, functional, and overall health complications.^(18,19)^ In terms of cognitive function, a significant proportion of ICU patients experience some degree of memory, attention, or executive function deterioration, with symptoms that sometimes linger for years after discharge.^(20,21)^ Therefore, the development of interventions from the ICU that impact the long-term cognitive status, quality of life and functionality is a priority.^(22)^

In this context, over last ten years, researchers have explored multidisciplinary rehabilitation strategies for early interventions in the ICU. Most of these studies have focused on physical therapy (PT) protocols that use early mobilization during the ICU stay to prevent neuromuscular dysfunction and progressively advance patients from mechanical ventilation to sitting, standing, and eventually walking.^(23-25)^

The first study that formally included OT as part of an early rehabilitation protocol in the ICU was performed by Schweickert et al.^(6)^ This trial included an intervention group that received progressive rehabilitation involving both physical and occupational therapists, beginning with passive mobility and advancing toward walking. Detailed descriptions of the physical and occupational therapy interventions are available.^(26)^ The focus of the OT intervention was training in ADLs and function training. For most of the sessions, the patients were able to participate in active mobility, sit on the edge of the bed, or simulate eating and grooming. Intubated patients were able to sit in an armchair during approximately one in three sessions, and the patients were able to participate in walking exercises during approximately 15% of the sessions. The primary endpoint for the study was functional independence in BADLs at discharge; the authors reported that the independence scores were significantly higher for the intervention compared with the control group (59% *versus* 35%, p = 0.02). After this study, other authors explored the feasibility, safety, and validity of the participation of OT in the ICU with similar results.^(7,26-28)^ OT interventions in the studies referred to above are shown in table 1.

**Table 1:** Occupational therapy interventions applied in intensive care unit patients

Activity	Objective	Description
Multisensory stimulation^(6,26)^	Increase alertness and prevent sensory deprivation	OT delivers the stimuli to the patient through different sensory channels
Positioning^(6,26)^	Prevent vicious positions and avoid loss of range of motion	OT uses devices for a comfortable position and support elements for the prevention of pressure ulcers, decreased range of motion and drop foot
Motor stimulation of the upper extremities^(6,7,26-28)^	Prevent muscle weakness acquired in the ICU	Activity in which the OT maintains active functions and strength of the upper extremities of patient movements through exercises
Cognitive stimulation^(28)^	Maintaining brain stimulation and connection with the environment	Intervention in which the OT retains active mental functions, such as orientation, attention, memory, calculus, problem solving, praxis, language, and visual perception, through stimulation protocols and dialogue with the patient.
Training in basic activities of daily living^(6,7,26-28)^	Maintain functional independence	Intervention in which the OT promotes independence in performing activities such as hygiene, grooming and feeding. In-patients with higher levels of independence are trained in costumes and transfers to structure the routine, maintain the level of functional independence and foster the feeling of usefulness.
Family involvement^(28)^	Promote interaction and family training	The OT holds meetings with the family to encourage their interactions with the patient during visiting hours and delivers material for use and strategies for cognitive stimulation.

OT - occupational therapy; ICU - intensive care unit.

Despite the progressive evidence supporting the role of OT as part of the rehabilitation team in the ICU, there are limitations on available data. All of the studies identified involve OTs as part of a multidisciplinary team where physical and occupational therapists work closely together, which makes it difficult to quantify the effect of the OT intervention alone, and the effectiveness of a specific pool of interventions.

### Delirium prevention and occupational therapy

Over the past 15 years, delirium in ICU patients has become a major topic in health care due to its high incidence and impact on long-term outcomes (morbidity, mortality, cognitive status, functional status, quality of life, and economic costs).^(1,2,5,29,30)^ Different strategies have been studied for the prevention and treatment of ICU *delirium*. They are grouped into non-pharmacological and pharmacological interventions.^(3,4)^ Several medications have been studied for the prevention and therapy of *delirium*, including different neuroleptics (i.e., haloperidol, risperidone, quetiapine, and olanzapine), dexmedetomidine, rivastigmine, dexamethasone and statins.^(3)^ Although recent guidelines on the use of sedatives, agitation and *delirium* in critically ill patients do not recommend the use of pharmacological prevention, a recent systematic review suggests a potential role for antipsychotics in surgical patients and dexmedetomidine in ventilated patients.^(3,31)^ New studies will help clarify the role of pharmacological prevention of ICU *delirium*.

Much attention has also given to different non-pharmacological interventions either individually or clustered into groups of measures for the prevention of *delirium*. Interest in these interventions comes from evidence from multicomponent programs for the prevention of delirium in elderly hospitalized patients.^(32-34)^ Indeed, recent guidelines of the American Geriatric Society suggest implementing multicomponent programs for *delirium* prevention in older patients.^(35,36)^

More than 10 different types of interventions for the non-pharmacological prevention of *delirium* in the ICU have been evaluated to date, as shown in table 2.^(4,37)^ Several of these interventions are part of the actions for which occupational therapists acquire skills during their professional education, including patient and health provider education, orientation, cognitive therapies and physical activities.

**Table 2:** Non-pharmacological strategies evaluated for the prevention of delirium in critical care^(4,37)^

1 - Modification of visual or auditory stimuli
- Noise reduction
- Earplugs
- Lighting control
- Eye mask
- Bright light therapy
- Music therapy
2 - Education
- To patient and family
- To health workers
3 - Orientation
4 - Cognitive therapy
5 - Physical therapy or exercise
- Early mobilization protocols
6 - Pharmacy protocol or review
7 - Awakening, breathing coordination and delirium monitoring
- ABCDE bundle implementation

ABCDE - **A**wakening and **B**reathing **C**oordination, **D**elirium Monitoring and Management, and **E**arly Mobility.

Protocols with physical and occupational therapy are strategies with evidence of efficacy. Schweickert et al.'s study included an a priori evaluation of *delirium* as a secondary endpoint for this trial and reported that the *delirium* duration was significantly reduced from 4 days in the control group to 2 days in the group that received the physical and occupational therapy interventions (p = 0.03).^(6)^ Similarly, the study of Needham et al., which included a before-after design to evaluate a quality improvement process, showed a significant increase in days without *delirium* in the patient group by including a team promoting early rehabilitation, including physical and occupational therapists.^(27)^

These findings and evidence supporting the efficacy of OT for other cognitive conditions prompted us to develop a clinical trial at our center for non-ventilated older adults admitted to the ICU. The preliminary and final results of this study have been recently published.^(38,39)^ The primary objective of this study was to evaluate the efficacy of an experimental non-pharmacological intervention (standard intervention plus early and intensive OT) in reducing the *delirium* incidence. A standard intervention was applied in both groups, which consisted of reorientation, mobility exercises, sensory deficit correction, environmental management, sleep protocols, and minimization of the use of drugs with the potential to trigger delirium. The experimental early/intensive OT intervention included multisensory stimulation, positioning, cognitive stimulation, and BADL training. A detailed description of the interventions is available online: http://www.medicina.uchile.cl/noticias/133590/terapia-ocupacional-disminuye-el-delirium-de-los-adultos-mayores.

The results showed a significantly lower incidence of delirium (3% *versus* 20%, p = 0.001), a higher level of functional independence (Functional Independence Measure (FIM) at discharge of 53 *versus* 31, p = 0.001), and better cognitive performance (cognitive FIM, p = 0,001) in the experimental compared to the control group after adjusting for age and education level.

According to these articles, the information available suggests the feasibility, safety and efficacy of OT in preventing *delirium*. However, there are important limitations that are relevant and should be reviewed. The main limitation is that there are few studies in this área. In most of the studies reviewed, delirium was a secondary outcome in studies exploring other primary outcomes. Further studies involving OT interventions in the ICU are necessary to assess the impact on *delirium* as a primary outcome. Additionally, most of the studies reviewed jointly evaluated the implementation of strategies that included physical and occupational therapy. Therefore, differentiating the specific impact of OT is very difficult. The only study that independently evaluated OT activity was the study of Alvarez et al., but this study was implemented in less severely ill patients who were unventilated and in intermediate care units. Moreover, the specific set of interventions applied for the prevention of *delirium* in ICU patients by occupational therapists are not defined because some differences exist in the described protocols. An economic evaluation of this intervention is not available because the available information to date only includes multi-professional rehabilitation programs in the ICU.^(40)^ Finally, evidence to document the long-term benefits of early OT interventions, including the impact on delayed cognitive and functional outcomes, is not available.

## CONCLUSION

To date, promising studies have suggested a role for occupational therapy in preventing *delirium* in the intensive care unit, but additional studies are needed to confirm and expand upon these findings. Under the potential benefits of the involvement of occupational therapists in the critical care team, particularly for the prevention of *delirium*, we suggest formally evaluating the incorporation of occupational therapist to the intensive care unit multiprofessional team. Specific interventions for implementation depend on the characteristics of each unit, especially its unique integration. Ideally, the interventions should be part of the early rehabilitation teams with physical and respiratory therapists. Older adults and ventilated patients will potentially benefit the most from early intervention.
